# The genetic puzzle of a SOD1-patient with ocular ptosis and a motor neuron disease: a case report

**DOI:** 10.3389/fgene.2023.1322067

**Published:** 2023-12-13

**Authors:** Veria Vacchiano, Flavia Palombo, Danara Ormanbekova, Claudio Fiorini, Alessia Fiorentino, Leonardo Caporali, Andrea Mastrangelo, Maria Lucia Valentino, Sabina Capellari, Rocco Liguori, Valerio Carelli

**Affiliations:** ^1^ IRCCS Istituto Delle Scienze Neurologiche di Bologna, UOC Clinica Neurologica, Bologna, Italy; ^2^ IRCCS Istituto Delle Scienze Neurologiche di Bologna, Programma di Neurogenetica, Bologna, Italy; ^3^ Dipartimento di Scienze Biomediche e Neuromotorie, Università di Bologna, Bologna, Italy

**Keywords:** *SOD1*, amyotrophic lateral sclerosis, *TBK1*, mitochondrial DNA, oligogenic inheritance

## Abstract

Amyotrophic lateral sclerosis (ALS) is a neurodegenerative disease with a complex genetic architecture, showing monogenic, oligogenic, and polygenic inheritance. In this study, we describe the case of a 71 years-old man diagnosed with ALS with atypical clinical features consisting in progressive ocular ptosis and sensorineural deafness. Genetic analyses revealed two heterozygous variants, in the *SOD1* (OMIM*147450) and the *TBK1* (OMIM*604834) genes respectively, and furthermore mitochondrial DNA (mtDNA) sequencing identified the homoplasmic m.14484T>C variant usually associated with Leber’s Hereditary Optic Neuropathy (LHON). We discuss how all these variants may synergically impinge on mitochondrial function, possibly contributing to the pathogenic mechanisms which might ultimately lead to the neurodegenerative process, shaping the clinical ALS phenotype enriched by adjunctive clinical features.

## 1 Introduction

Amyotrophic lateral sclerosis (ALS) is a fatal neurodegenerative disease conventionally classified as familial or sporadic. However, besides this simple subdivision, the genetic architecture underlying the disease’s pathogenesis is highly complex, showing monogenic, oligogenic, and polygenic inheritance, with variable gene penetrance and heritability (Goutman et al., 2022). Monogenic familial amyotrophic lateral sclerosis accounts for 10%–15% of affected individuals, albeit with incomplete penetrance (Ryan et al., 2019). In the remaining 85%, large genome-wide association studies (GWAS) have been useful to identify rare or private variants that might act as risk factors and/or disease modifiers, thus modulating phenotypic presentation (Ryan et al., 2019). Nowadays, ALS’ pathogenesis is considered as a multi-step disease process, where multiple hits, both genetic and environmental, are needed to develop the disease (Al-Chalabi et al., 2014). Furthermore, several observations proved that the burden of multiple genetic rare variants might trigger the degenerative process, modulating also the clinical phenotype (van Blitterswijk et al., 2012). Recently, a large study on ALS (van Rheenen et al., 2021) pointed to the burden of multiple risk factors disclosed in the nuclear genome but missed to consider the possible impact of mitochondrial DNA (mtDNA) variation, which is frequently neglected but may contribute to the pathogenesis or modulate the phenotype also in ALS. As an example, we recently reported the unique association of ALS and Leber’s hereditary optic neuropathy (LHON) in two unrelated patients who had a late onset ALS with rapid diseases course, speculating that mtDNA might have contributed as a possible risk factor or disease modifier in ALS (Amore et al., 2023). Here we describe an atypical motor neuron disease in a *SOD1* patient carrying an additional variant in *TBK1* gene and the LHON-associated m.14484T>C variant in the mtDNA. This case supports the oligogenic nature of ALS and further rises questions on the possible contributory role of mtDNA variation in the ALS pathogenesis and clinical expression.

## 2 Clinical case

A 71-years-old man came to our attention for the progressive onset of weakness in his legs, started 3 years before, and worsened in the past year, leading to the use of aids in walking. Since the age of 58, he also reported progressive bilateral eyelid droop, without clear daily fluctuation. His past history was relevant for hypertension under pharmacological treatment. From the age of 54, he also suffered from a progressive hearing loss. His audiometry revealed an auditory loss especially for high frequencies, consistent with a sensorineural deafness (Supplementary Figure S1).

In his family history, he had a brother with progressive hearing loss (started at 55 years), and unspecified gait disturbances associated with increased creatin kinase (CK) levels. The patient did not report any other neurological disease recurring in his family.

In the suspicion of myasthenia gravis, at the age of 69 years the patient had performed a single fiber electromyography (EMG) of the frontalis muscle, which resulted negative. In the same period, he was also treated with oral steroid therapy (prednisone at the dosage of 25 mg/die) for several months, subsequently interrupted for inefficacy.

Our neurological examination revealed bilateral hypoacusia, bilateral severe ocular ptosis without deficit in extrinsic ocular movements. Exacerbation of ptosis after effort or repeated closing of the eyes was absent. The patient also presented hyposthenia and hypotrophy in the lower limbs, prevalent in the distal compartment of the right leg (tibialis anterior MRC scale 3/5, gastrocnemius MRC 3/5, longus and brevis peroneus MRC 3/5, flexor hallucis longus MRC 3/5, iliopsoas MRC 4/5, biceps femoris MRC 4/5). The left lower limb was slightly weak only in the proximal side (iliopsoas MRC 4/5). Deep tendon reflexes were diffusely reduced. Spasticity, Babinski or Hoffman signs were not observed. Gait showed right drop foot with steppage.

Due to the association of hypoacusia and bilateral ptosis, a mitochondrial disorder was suspected.

Routine blood exams only showed an increase of CK (367 U/L, n.v. < 170). Antibodies against acetylcholine receptor and anti-MUSK were negative.

**FIGURE 1:**
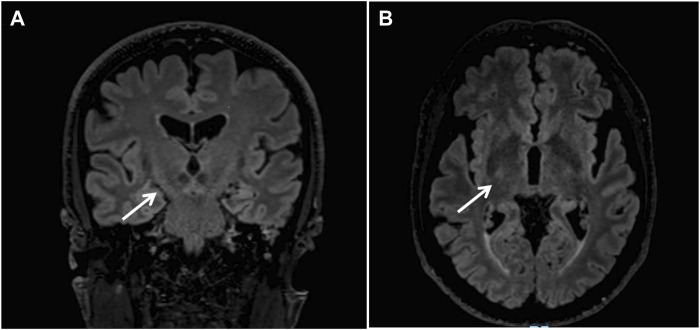
Brain Magnetic Resonance revealed bilateral fronto-parietal atrophy; coronal **(A)** and axial **(B)** FLAIR-T2-weighted sequences displayed hyperintensity of the cortico-spinal tracts (arrows).

Brain Magnetic Resonance (MR) revealed bilateral fronto-parietal atrophy and mild signs of microangiopathy. FLAIR-T2-weighted sequence displayed hyperintensity of the cortico-spinal tracts (Figures 1A, B). Spectroscopy did not reveal pathological lactate in ventricles.

A complete spinal MR with gadolinium was unremarkable.

A comprehensive ophthalmologic examination revealed normal visual acuity and fondus oculi. The optic coherence tomography (OCT) failed to show any abnormalities.

Similarly, the cerebrospinal fluid (CSF) analysis revealed normal proteins and cells levels. IgG and blood-brain barrier indexes were in the normal range, oligoclonal bands were not detected.

Nerve conduction studies, quantitative EMG and repetitive nerve stimulation were performed. Motor conduction study of the left ulnar nerve, bilateral tibial and peroneal nerves only showed a severe decrease of the amplitude of compound motor action potential (CMAP) in the right tibial and peroneal nerves, while sensory conduction study of left ulnar, bilateral sural and peroneal nerves was in the normal range. Quantitative EMG analysis revealed the presence of subacute neurogenic changes in the thoracic region (T5 myotome) and in lower limbs (right > left), in particular in right vastus medialis, left gastrocnemius and bilateral tibialis anterior muscles. Genioglossus, right biceps brachialis and left first dorsal interosseous were completely normal. The repetitive stimulation at 3–5 Hz of bilateral facial nerves recording from nasalis and mentalis muscles, such as of the ulnar nerve recording from the abductor digiti minimi muscle did not show a significative decrement or increment of the amplitude of the CMAP. The stimulation before and after a prolonged effort (20 s) of the left ulnar and right peroneal nerves did not show significative changes in the CMAP amplitude.

These findings were not consistent with a sensory-motor polyneuropathy or a dysfunction of the neuromuscular junction, but the presence of subacute neurogenic changes raised the suspicion of a motor neuron disease.

**FIGURE 2:**
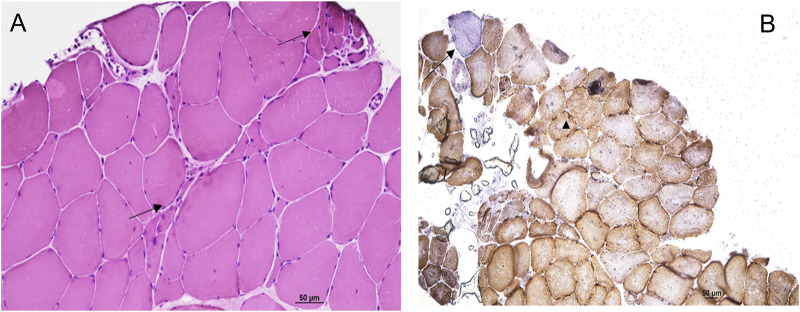
Hematoxilin Eosin staining **(A)** showed neurogenic changes with small groups of atrophic fibers (arrows). Cytochrome C Oxidase/Succinate dehydrogenase double staining **(B)** showed a few fibers displaying subsarcolemmal enhancement (head of arrow) and a single COX negative fiber (arrow).

The tibialis anterior muscle biopsy confirmed the presence of neurogenic changes with small groups of atrophic fibers (Figure 2A). Cytochrome C Oxidase/Succinate dehydrogenase double staining (Figure 2B) showed a few fibers displaying subsarcolemmal enhancement and a single COX negative fiber, both still consistent with secondary and possibly age-dependent abnormalities and not configuring a mitochondrial myopathy by themselves.

Genetic analysis, through Whole Exome Sequencing (WES) with an *in silico* panel analysis, was initially focused on the fronto-temporal dementia (FTD)-ALS related genes (*SOD1, FUS, TARBP, SEXT, ANXA11, CHCHD10, DTCN1, FIG4, HNRNPA1, KIF5A, MATR3, NEK1, OPTN, PFN1, SIGMAR1, SPG11, SQSTM1, TUBA4A, UBQLN2, VAPB, VCP*), and revealed the presence of the heterozygous variant c.412A>G (p.Thr138Ala) in the *SOD1* gene (NM_000454.5), and the heterozygous variant c.422T>C (p.Ile141Thr) in the *TBK1* gene (NM_013254.4). The presence of expanded alleles in the *C9Orf72* gene was also excluded.

The heterozygous p.Thr138Ala variant in the *SOD1* gene (OMIM*147450), which was not reported in the gnomAD v.2.1.1 database, was classified as pathogenic (PP5, PP3, PM2, PM5, PM1, PP2 criteria) according to the American College of Medical Genetics (ACMG) guidelines (Richards et al., 2015). Relevantly, the p. Thr138Ala could be a likely founder variant, since it was reported in three other Italian patients with ALS, supporting its pathogenic role (Visani et al., 2011; Origone et al., 2012; Zhang et al., 2016).

The heterozygous p.Ile141Thr in the *TBK1* gene (OMIM*604834) was a very rare variant (1/250,400 alleles in gnomAD) and was classified as hot VUS (PP3, PM2) according to the ACMG guidelines. This gene has been associated to ALS and/or FTD with an autosomal dominant inheritance (MIM#616439).

Therefore, the patient presented a clinical picture characterized by the involvement of lower motor neurons restricted to the lumbosacral and thoracic regions, thus configuring a clinical diagnosis of progressive muscular atrophy (PMA), still recognized as ALS according to the Gold coast criteria (Shefner et al., 2020), and confirmed by the presence of a pathogenic variant in the ALS-causative *SOD1* gene.

However, due to the progressive bilateral ptosis, atypical for a motor neuron disease, we also screened a panel of genes associated to chronic progressive external ophthalmoplegia (CPEO) (*ABAT, AFG3L2, C10orf2, DGUOK, DNA2, DNM1L, FBXL4, GFER, MFN2, MGME1, MPV17, OPA1, POLG, POLG2, RNASEH1, RRM2B, SLC25A10, SPG7, SUCLA2, SUCLG1, TFAM, TK2, TOP3A, TYMP*) that resulted negative as well as a panel of 176 genes associated with non-syndromic hearing loss and deafness (genes are listed in the Supplementary Materials).

Finally, to further investigate the occurrence of ptosis suspecting a mitochondrial myopathy, we analyzed the mtDNA extracted from skeletal muscle. Quantitative testing for pathologic accumulation of single/multiple macrodeletions was normal and the mtDNA copy number assessment showed an increase of around 30% compared to controls. The complete mtDNA sequencing revealed the presence of the Leber’s hereditary optic neuropathy (LHON) associated homoplasmic variant m.14484T>C/*MT-ND6* on a H1a3c haplogroup background.

Neurofilament light chain levels in the CSF were also investigated and were significantly increased (5,822 pg/mL, n.v. 340–650).

The patient’s clinical conditions continued to progress over the years: at a 2-year follow-up he was not able to stand or walk autonomously anymore, and he also showed a moderate hypotrophy and weakness in the upper limbs with functional deficits, without bulbar involvement. No other new clinical signs of mitochondrial disease were observed. The patient has been enrolled in the expanded access program for Tofersen, and had this treatment for 4 months (follow-up ongoing), referring clinical stability.

## 3 Discussion

Ocular ptosis is not a classic symptom of ALS, although it has been rarely reported (Pinto and de Carvalho, 2008; De Marchi et al., 2018; Shindo et al., 2020). Intriguingly, De Marchi et al. reported two siblings with a history of progressive ptosis without ocular movement impairment, diagnosed with a bulbar ALS with rapid progression (De Marchi et al., 2018). Thus, notwithstanding the rarity, our case is not unprecedented. To disclose the possible genetic cause, we first aimed at screening the major genes associated with ALS and FTD, as well as searching for expansion in *C9Orf72*, revealing a combination of two different genetic variants of interest affecting the *SOD1* and *TBK1* genes respectively.

To further refine the genetic investigation, in consideration of the ptosis and sensorineural deafness affecting our patient pointing to a possible mitochondrial disease, we also screened mtDNA extracted from the muscle biopsy. Analyses for mtDNA single/multiple deletions was within normal range. Sequencing the entire mtDNA revealed as *incidental* finding the homoplasmic pathogenic variant m.14484T>C/*MT-ND6*, on a haplotype H1a3c background. The copy number was higher than control range, in agreement with the compensatory mechanism already described in patients carrying LHON pathogenic variants (Giordano et al., 2014) and congruently to the occasional subsarcolemmal increase of COX/SDH staining. The m.14484T>C/*MT-ND6* variant is causative of LHON, but was never associated with mitochondrial myopathies characterized by ptosis/PEO, and therefore cannot explain by itself the ocular ptosis of our patient. Furthermore, our patient did not show any signs of optic neuropathy, and his family history was negative for cases suggestive of LHON on the maternal lineage. This is not surprising, as it is well established that the m.14484T>C/*MT-ND6* variant expresses the LHON phenotype principally in the context of the haplogroup J (Hudson et al., 2007) and has been frequently found in population screenings without being linked to LHON (Yonova-Doing et al., 2021). In fact, as recently highlighted, the LHON m.11778G>A/*MT-ND4* and m.14484T>C/*MT-ND6* variants have been both found to be present in 1 every 800–1,000 individuals from normal populations, associated with low penetrance mtDNA backgrounds such as haplogroups H and U (Mackey et al., 2023; Watson et al., 2023), thus reinforcing the idea that deleterious mtDNA variants may be incidentally found when investigating patients for a neurodegenerative disorder.

Overall, our patient presented a clinical picture consistent with a PMA, characterized by a prevalent/selective dysfunction of the lower motor neurons. This entity, although previously not recognized as ALS by both Revised El Escorial (Brooks et al., 2000) and Awaji criteria (de Carvalho et al., 2008), has been categorized as “ALS” in the most recent Gold coast criteria (Shefner et al., 2020), due to an increasing body of evidence suggesting a subclinical involvement of corticospinal tracts in both neuropathological (Ince et al., 2003) and imaging (van der Graaff et al., 2011; Rosenbohm et al., 2016) studies on patients with PMA. In this line, it is not surprising that brain MRI of our patient revealed T2/FLAIR hyperintensities of the corticospinal tracts, which might be interpreted as a qualitative subclinical sign of UMN involvement (Rizzo et al., 2020).

Furthermore, the PMA phenotype with the relatively long disease duration in our patient is quite consistent with the presence of the pathogenic variant in *SOD1* gene, already described in individuals with a spinal onset, absence of bulbar involvement and a slow disease progression, with a variable age at onset (mean 53 years, ranging from 35 to 73 years) (Visani et al., 2011; Origone et al., 2012; Zhang et al., 2016). On the other hand, the co-occurrence of another variant in an ALS-FTD gene, the *TBK1* gene, is consistent with the oligogenic and complex background underlying both sporadic and familial ALS (Goutman et al., 2022). *TBK1* has been reported to contribute to around 1.3% of ALS, 3%–4% of ALS‐FTD, and less than 1% of FTD with TDP‐43 pathology (Nguyen et al., 2018). Among patients carrying a mutation in *TBK1* gene, more than 50% were diagnosed with ALS, 14% with FTD‐ALS, 18% with FTD, and 1.3% with AD (Yu et al., 2019). The clinical manifestations are highly heterogeneous, and intrafamilial and interfamilial heterogeneity were also reported in patients carrying the same variants (Freischmidt et al., 2015). The most common initial symptoms included limb weakness, cognitive deficits, and bulbar signs. This extreme heterogeneity makes genetic-phenotype correlations quite challenging.

Furthermore, the uncertain role of the mtDNA m.14484T>C/*MT-ND6* pathogenic variant deserves a few more comments. We previously reported the unprecedented occurrence in two unrelated patients with ALS of another LHON homoplasmic mutation, the most common m.11778G>A/*MT-ND4* change, associated with optic atrophy in only one of the two patients. We speculated that the mtDNA could have played a role as disease modifier in these patients, as they were characterized by late-onset ALS and rapid course to death (Amore et al., 2023). Our current findings in this other patient remark that mtDNA variants in ALS are probably more frequent than expected and deserve to be systematically explored in conjunction with the nuclear genome analysis, to better evaluate their possible role in the disease pathogenesis, penetrance and clinical evolution. In support, rare cases of ALS patients with causative mtDNA mutations and clear signs of mitochondrial disease have also been reported (Hirano et al., 2008).

Remarkably, all genes found in our patient are known to play an essential role in mitochondrial function. First, the Cu/Zn superoxide dismutase 1 protein (SOD1) is an abundantly expressed antioxidant enzyme that exists as a homodimer and localizes to the cytosol, but also in the intermembrane mitochondrial space, whereas the manganese superoxide dismutase 2 (SOD2) is in the mitochondrial matrix (Vijayvergiya et al., 2005). Human SOD1 mutant proteins seem to gain a toxic property or function, rather than having diminished O2 − scavenging activity (Pasinelli et al., 2004; Martin, 2010; Bille et al., 2013). The toxicity is due to the misfolded aggregates of both mutant and wild-type SOD1 that disrupt mitochondrial respiration, Ca2+ homeostasis and turnover, leading to increased ROS generation, autophagy/mitophagy capacity decrease and more detrimental events such as the release of apoptotic signals promoting the opening of mitochondrial membrane permeability pore (Martin, 2010). Indeed, it is already known that in the SOD1-G93A mouse model mitochondria appeared with dilated and disorganized cristae, both in the axons and dendrites of motor neurons at onset of disease (Vande Velde et al., 2011). Changes in mitochondrial morphology, such as swelling or enlargement, were also found in soma, proximal axons and motor nerve terminals in tissue from ALS patients (Sasaki and Iwata, 2007), which result in an impairment of their axonal transport in motor neurons (De Vos et al., 2007). Since morphological abnormalities in mice appeared before the onset of symptoms of motor neuron degeneration, it has been postulated that mitochondria impairment may play a key role in starting motor neuron degeneration in ALS (Zhou et al., 2010).

On the other hand, the NF-kappa-B-activating kinase encoded by the *TBK1* gene plays a critical role in several cellular pathways, including selective clearance of mitochondria and regulation of inflammation. Indeed, this kinase binds to and phosphorylates a number of proteins involved in innate immunity and autophagy, including optineurin (OPTN) and p62, both of which have been implicated in ALS. In details, TBK1-OPTN axis targets damaged mitochondria for degradation via PINK1/parkin-mediated mitophagy (He et al., 2017). Functional studies revealed that mutations disrupting the structure of the protein impair the recruitment of TBK1 to damaged mitochondria, inhibiting the mitophagy process (Richter et al., 2016). Moreover, primary neurons expressing TBK1 mutations showed higher baseline levels of mitochondrial stress and a reduce competence to control induced oxidative damage, both of which mechanisms may contribute to neurodegeneration (Harding et al., 2021).

To conclude, as primary mtDNA defects such as the m.14484T>C/*MT-ND6* variant are well known to impair the activity of complex I, leading to a decrease in ATP synthesis and an increasing generation of ROS (Carelli et al., 1999; Baracca et al., 2005), we propose as plausible that all variants identified in this patient may contribute to mitochondrial dysfunction and motor neuron degeneration. However, the clinical picture remains atypical for both motor neuron and mitochondrial diseases, also considering the lack of clearcut mitochondrial abnormalities in muscle biopsy and at MR spectroscopy.

This case illustrates how multiple nuclear and mitochondrial variants may ultimately contribute to the neurodegenerative process, with a leading role for mitochondrial dysfunction in ALS pathogenesis. A systematic parallel analysis of nuclear encoded risk factors for ALS in conjunction with mtDNA sequence analysis is warranted in large cohort studies to fully clarify this possible missing genetic contribution to ALS pathogenesis.

## Data Availability

The data presented in the study are deposited in the BioProject database, accession number BioProject ID: PRJNA1047484.
