# Supplementary material for: The genetic puzzle of a SOD1-patient with ocular ptosis and a motor neuron disease: a case report
Source: Front Genet. 2023 Dec 13;14:1322067. doi: 10.3389/fgene.2023.1322067 (PMC10751346; doi:10.3389/fgene.2023.1322067)
Supplement: Supplementary file 1 [file DataSheet1.docx]

Supplementary Material

# Supplementary Data

**Genes associated with non-syndromic hearing loss and deafness investigated and excluded in our patient are listed below:**

*ABHD12; ACTG1; ADGRV1; AIFM1; ALMS1; AP1S1; ATP6V1B1; ATP6V1B2; BCS1L; BSND; CABP2; CCDC50; CDC14A; CDH23; CEACAM16; CEP250; CEP78; CHD7; CIB2; CISD2; CLDN14; CLDN9; CLPP; CLRN1; COCH; COL11A1; COL11A2; COL2A1; COL4A5; COL9A1; COL9A2; CRYM; DFNA5; DFNB59; DIAPH1; DMXL2; DNMT1; DSPP; EDN3; EDNRB; EPS8; EPS8L2; ESPN; ESRRB; EYA1; EYA4; FDXR; FGF3; FOXI1; GATA3; GGPS1; GIPC3; GJB2; GJB3; GPSM2; GREB1L; GRHL2; GRXCR1; HAAO; HARS2; HOXA2; HSD17B4; ILDR1; KARS; KCNE1; KCNJ10; KCNJ16; KCNQ1; KCNQ4; KIT; LARS2; LHFPL5; LMX1A; LOXHD1; LRTOMT; MARVELD2; MASP1; MITF; MN1; MPZL2; MSRB3; MYH14; MYH9; MYO15A; MYO3A; MYO6; MYO7A; OGDHL; OPA1; OSBPL2; OTOA; OTOF; OTOG; OTOGL; P2RX2; PAX2; PAX3; PBX1; PCDH15; PDZD7; PLS1; PNPT1; POU3F4; POU4F3; PRPS1; PTPRQ; RDX; RNF220; S1PR2; SALL1; SALL4; SERAC1; SERPINB6; SGPL1; SIX1; SLC12A2; SLC17A8; SLC26A4; SLC26A5; SLC4A11; SLC52A2; SLC52A3; SLITRK6; SMPX; SOX10; SOX2; SPATA5; SPATA5L1; STRC; SYNE4; TBC1D24; TECTA; TIMM8A; TMC1; TMIE; TMPRSS3; TPRN; TRIOBP; USH1C; USH1G; USH2A; USP48; WFS; WHRN.*

# Supplementary Figure 1

# Audiometry showed an auditory loss especially for high frequencies, consistent with a sensorineural deafness.


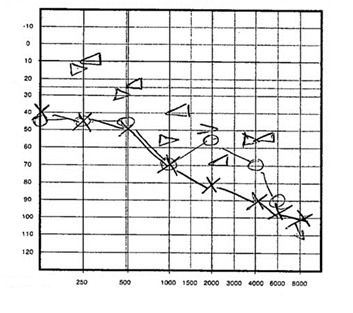


**Frequencies (Hz)**

**Auditory loss (dB)**
